# New anthropometry-based age- and sex-specific reference values for urinary 24-hour creatinine excretion based on the adult Swiss population

**DOI:** 10.1186/s12916-015-0275-x

**Published:** 2015-02-27

**Authors:** Valentina Forni Ogna, Adam Ogna, Philippe Vuistiner, Menno Pruijm, Belen Ponte, Daniel Ackermann, Luca Gabutti, Nima Vakilzadeh, Markus Mohaupt, Pierre-Yves Martin, Idris Guessous, Antoinette Péchère-Bertschi, Fred Paccaud, Murielle Bochud, Michel Burnier, on behalf of the Swiss Survey on Salt Group

**Affiliations:** Service of Nephrology and Hypertension, University Hospital of Lausanne (CHUV), Rue du Bugnon 17, 1011 Lausanne, Switzerland; Department of Internal Medicine and Nephrology, Regional Hospital, Locarno, Switzerland; Institute of Social and Preventive Medicine (IUMSP), Lausanne University Hospital, Lausanne, Switzerland; Service of Nephrology, Department of Specialties, University Hospital of Geneva, Geneva, Switzerland; University Clinic for Nephrology, Hypertension and Clinical Pharmacology, Inselspital, Bern University Hospital and University of Bern, Bern, Switzerland; Unit of Population Epidemiology, Geneva University Hospitals, Geneva, Switzerland; Department of Community Medicine and Primary Care and Emergency Medicine, University Hospital of Geneva, Geneva, Switzerland

**Keywords:** 24-hour urinary collection, Urinary creatinine excretion, European population, Prediction equation, Reference values, Normograms

## Abstract

**Background:**

Urinary creatinine excretion is used as a marker of completeness of timed urine collections, which are a keystone of several metabolic evaluations in clinical investigations and epidemiological surveys.

The current reference values for 24-hour urinary creatinine excretion rely on observations performed in the 1960s and 1970s in relatively small and mostly selected groups, and may thus poorly fit to the present-day general European population.

The aim of this study was to establish and validate anthropometry-based age- and sex-specific reference values of the 24-hour urinary creatinine excretion on adult populations with preserved renal function.

**Methods:**

We used data from two independent Swiss cross-sectional population-based studies with standardised 24-hour urinary collection and measured anthropometric variables. Only data from adults of European descent, with estimated glomerular filtration rate (eGFR) ≥60 ml/min/1.73 m^2^ and reported completeness of the urinary collection were retained. A linear regression model was developed to predict centiles of the 24-hour urinary creatinine excretion in 1,137 participants from the Swiss Survey on Salt and validated in 994 participants from the Swiss Kidney Project on Genes in Hypertension.

**Results:**

The mean urinary creatinine excretion was 193 ± 41 μmol/kg/24 hours in men and 151 ± 38 μmol/kg/24 hours in women in the Swiss Survey on Salt. The values were inversely correlated with age and body mass index (BMI).

Based on current reference values (177 to 221 μmol/kg/24 hours in men and 133 to 177 μmol/kg/24 hours in women), 56% of the urinary collections in the whole population and 67% in people >60 years old would have been considered as inaccurate.

A linear regression model with sex, BMI and age as predictor variables was found to provide the best prediction of the observed values and showed a good fit when applied to the validation population.

**Conclusions:**

We propose a validated prediction equation for 24-hour urinary creatinine excretion in the general European population, based on readily available variables such as age, sex and BMI, and a few derived normograms to ease its clinical application. This should help healthcare providers to interpret the completeness of a 24-hour urine collection in daily clinical practice and in epidemiological population studies.

## Background

Creatinine is a break-down product of the intracellular creatinine precursors creatine and creatine- phosphate. Most of the creatinine is derived from nonenzymatic processes occurring in skeletal muscle at a fairly constant rate, depending on muscle mass [1,2]. Creatinine is freely filtered out of the blood by the glomerulus and is neither reabsorbed nor metabolised by tubular cells. Proximal tubular secretion accounts for 10% to 20% of total urinary creatinine in patients with normal renal function, a percentage that rises as the glomerular filtration rate falls [2,3]. Creatinine excretion in the urine occurs at a fairly constant rate over 24 hours and is, therefore, used as a reference comparator for analysis performed on spot urine and timed urine samples.

Measurement of urinary creatinine excretion in the clinical and research fields has multiple purposes: 1) to measure creatinine clearance as a surrogate of the glomerular filtration rate [2]; 2) to estimate average 24-hour excretion rates of several solutes including electrolytes and proteins from spot urine samples, using the respective ratios to urinary spot creatinine concentration [4-7]; 3) to assess lean body mass [1,8]; and 4) to check the completeness of 24-hour urine collections [9,10]. The latter can be estimated from knowledge of the normal rate of creatinine excretion, which is equal to creatinine production in the steady state. In longitudinal trials reporting completeness criteria of the 24-hour urine collection, intra-individual variation in creatinine output of repeated collections was used as a quality assurance tool to assess the accuracy [11,12]. However, in the case of a single patient’s measure and in large scale epidemiologic studies, the identification of over- and under- collection of 24-hour urine samples remains a crucial but difficult task [10,13].

The accuracy of a 24-hour urine collection is mainly evaluated by computing the creatinine excretion per kilogram body weight over 24 hours. According to the general nephrology references, the daily creatinine excretion should be 177 to 221 μmol/kg (20 to 25 mg/kg) in men and 133 to 177 μmol/kg (15 to 20 mg/kg) in women [14]. These reference values rely on observations performed in the 1960s and 1970s in relatively small and mostly selected groups (for example, hospitalised patients) [15-18].

The European population has markedly changed over the last 50 years, aging progressively and showing a steadily increasing prevalence of obesity [19-21]. Both these factors impact on the body composition and on the proportion of muscle mass, the main determinant of urinary creatinine excretion. The adequacy of the classical reference values to the European general population should, therefore, be questioned.

The aim of the present study was to establish anthropometry-based age- and sex-specific reference values of the urinary 24-hour creatinine excretion in a large Swiss population-based sample of European descent with preserved renal function and to validate these values in an independent population-based sample.

## Methods

### Study design

We used the data collected during the Swiss Survey on Salt (SSS) [22] as a derivation set to fit the prediction model. SSS is a cross-sectional, population-based survey including people 15-years old and over. The data from a second independent population-based study, the Swiss Kidney Project on Genes in Hypertension (SKIPOGH) [23,24], were used as a validation set to evaluate the developed model.

### Study population

SSS was promoted by the Swiss Federal Office of Public Health (BAG) within the context of the national program on Nutrition and Movement. It aimed primarily at estimating the dietary salt intake and hypertension prevalence. The sample population was selected to represent the three linguistic regions of Switzerland (French, German and Italian) and to be equally distributed in four age categories (15 to 30, 30 to 45, 45 to 60 and over 60 years) for each sex. Recruitment began in January 2010 and ended in August 2011. Details of the SSS study design have been published previously [22]. The SSS complied with the Declaration of Helsinki and was approved by the local Institutional Ethics Committees. All participants gave written informed consent.

The SKIPOGH study is a family-based longitudinal study exploring the role of genes, kidney hemodynamics and the environment on blood pressure regulation and hypertension. Participants were recruited in the cantons of Bern and Geneva, and the city of Lausanne. Details of the SKIPOGH study design have been published previously [23]. The SKIPOGH study complied with the Declaration of Helsinki and was approved by the local Institutional Ethics Committees. All participants gave written informed consent.

In both populations, only data from people of European descent ≥18-years old, with an estimated glomerular filtration rate (eGFR) ≥60 ml/min/1.73 m^2^ according to the Chronic Kidney Disease Epidemiology Collaboration (CKD-EPI) equation [25] and with complete urine collection (as defined below) were retained for the present analysis.

### Clinical data

In both studies participants performed a 24-hour urine collection following oral and standardised written instructions. At reception, urine volume was measured, and sampled in small aliquots and immediately frozen (−20°C in SSS and −80°C in SKIPOGH) at each study center. In order to assess reproducibility of urine collection and urinary creatinine excretion, a randomly selected sub-sample of participants in the SSS provided 24-hour urine collections on two consecutive days.

Incomplete urine collections - defined as urine loss during the collection time (self-reported by the participant, N = 20 in SSS), collections with urine volume <300 ml (N = 3) and/or lasting <20 hours (N = 5) - and over-collections, defined as urinary creatinine >400 μmol/kg/24 hours (N = 0), were excluded from the present analysis, in line with the criteria adopted in previous large international studies [26].

Body weight was measured in light indoor clothing to the nearest 100 g using a medical scale and height was measured to the nearest centimeter using a wall-mounted stadiometer. In SSS, an optional non-fasting blood sample was collected whereas in SKIPOGH, a fasting blood sample was collected in all participants.

### Laboratory analysis

Urine and blood samples were analysed in the Central Chemical Laboratory of Lausanne University Hospital (CHUV, Lausanne, Vaud, Switzerland) for all centers involved in SSS and for the Lausanne sample in SKIPOGH. Analyses were done in the Central Chemical Laboratory of Geneva and Bern University Hospitals for the SKIPOGH Geneva and Bern samples, respectively. Serum and urine creatinine were measured using the kinetic colorimetric compensated Jaffe method, as reported by the manufacturer, Roche Modular P System (Roche Diagnostics, Mannheim, Germany). In all laboratories, creatinine was measured using isotope dilution mass spectrometry (IDMS)-traceable methods. We compared the creatinine measurement of 20 fresh (all measurements conducted on the same day) serum and urine samples across the three laboratories involved in SKIPOGH and found a Lin’s concordance correlation coefficient ranging between 0.87 and 0.92 for serum creatinine and equal to 1 (perfect correlation) for urine creatinine, which denotes excellent inter-laboratory reproducibility.

### Definition of covariates

The CKD-EPI formula was used to estimate the glomerular filtration rate (eGFR) [25]. In SSS, diabetes was considered present when a participant reported to take diabetes medications. In SKIPOGH, diabetes was considered present when reported or treated or when fasting glycemia was ≥7 mmol/L. In both studies, hypertension was defined as mean office blood pressure ≥140/90 mm Hg or treated hypertension.

### Statistical analysis

Statistical analyses were performed using STATA 12.0 (StataCorp, College Station, Texas, USA) and R 3.1.0 (R Core Team, 2014, R Foundation for Statistical Computing, Vienna, Austria). Quantitative variables with normal distribution were expressed as mean ± standard deviation and categorical ones as percentage of participants. We compared continuous and categorical variables in men and women using t-test and chi-square tests. Statistical significance was established for *P* <0.05.

We used linear regression modelling to predict the 24-hour urinary creatinine excretion per body weight (expressed in μmol/kg/24 hours) in the derivation population (SSS), using the measured anthropometric variables as candidate factors and adjusting for the study centres. We developed various models including gender, age, weight, BMI, and explored the existence of interactions between the factors. The models were compared in their goodness of fit, selecting as the final model the one that minimised the Bayesian Information Criterion (BIC). Assuming normality of urinary creatinine excretion distribution (Kolmogorov-Smirnov test, *P* = 0.61), the standard deviation of the model’s residuals was used to predict any centile of the distribution: the α centile is given by the fitted value plus the α quantile of a standard normal distribution times the residuals standard deviation.

For the external validation of this predictive model we used the data from the SKIPOGH study. In the absence of a validated gold standard for the adequacy of the 24-hour urinary collection, the predicted centile of each urine creatinine excretion was computed using the developed model and the uniformity of their distribution was verified with a Kolmogorov-Smirnov test. We, finally, computed the reclassification effect of the new model compared to the current reference values, using the 10th to 90th centile range as an example of cut-off to define adequate urinary collection.

## Results

### Study population

A total of 1,550 participants completed the SSS study. A total of 1,137 (550 men and 587 women) were included in the present analysis. We excluded 413 people because of age <18 years (n = 39), non-Caucasian race (n = 115), eGFR <60 ml/min/1.73 m^2^ (n = 99), impossibility to assess renal function because blood sampling was refused (n = 132) or incomplete 24-hour urinary collection (n = 28). Anthropometric characteristics and serum creatinine showed significant differences between sexes, whereas eGFRs estimated by the CKD-EPI equation were similar (Table 1).

**Table 1:** **Descriptive data of the derivation (SSS) and validation population (SKIPOGH), by sex**

	**SSS**		**SKIPOGH**	
**Variables**	**Men**	**Women**	**Men**	**Women**
Number	550	587	473	521
Age (years)	48.1 (17.2)	46.2 (16.8)	47.0 (17.6)	47.6 (16.7)
Weight (kg)	81.7 (14.2)	66.3 (13.2)^a^	81.5 (12.9)	65.4 (12.6)^a^
Height (cm)	176.5 (7.0)	164.6 (6.7)^a^	177.4 (6.8)^d^	164.9 (6.4)^a^
BMI (kg/m^2^)	26.2 (4.1)	24.5 (4.8)^a^	25.9 (4.1)	24.1 (4.6)^a^
Hypertension (Number, %)	180 (32.8)	100 (17.0)^a^	130 (27.5)	96 (18.5)^a^
Diabetes (Number, %)	40 (7.3)	39 (6.6)	30 (6.3)	13 (2.5)^b^
Plasma creatinine (μmol/L)	86.6 (11.5)	69.0 (8.8)^a^	80.2 (11.0)^c^	65.8 (9.3)^a^ ^c^
eGFR CKD-EPI (ml/min/1.73 m^2^)	92.1 (15.4)	92.0 (16.6)	99.0 (16.7)^c^	95.5 (15.7)^a^ ^c^
Duration of urine collection (minutes)	1442 (65)	1447 (78)	1442 (81)	1436 (76)^d^
Volume of urine collection (ml)	1928 (876)	2072 (922)	1698 (756)^c^	1719 (702)^c^
Urinary creatinine excretion (μmol/24 hours)	15620 (3768)	9821 (2518)^a^	15651 (3413)	10273 (2383)^a^ ^c^
Urinary creatinine excretion (μmol /kg/24 hours)	193 (41)	151 (38)^a^	194 (42)	161 (40)^a^ ^c^

Data are expressed as mean (SD) unless otherwise specified. ^a^ *P* <0.001 compared to men; ^b^ *P* <0.01 compared to men; ^c^ *P* <0.001 compared to SSS; ^d^ *P* <0.05 compared to SSS. SSS: Swiss Survey on Salt.

In the SKIPOGH study, 994 people were considered as the validation population for the present analysis, after excluding 60 people for incomplete urine collection and 3 for over-collection. This population is also characterised in Table 1. In this population, mean blood creatinine values were slightly lower than in SSS, whilst 24-hour urinary volume was higher in SSS, reflecting the regional differences between participants, with German speaking people having higher urine volume than French speaking ones.

### Urinary creatinine excretion

**Figure 1:**
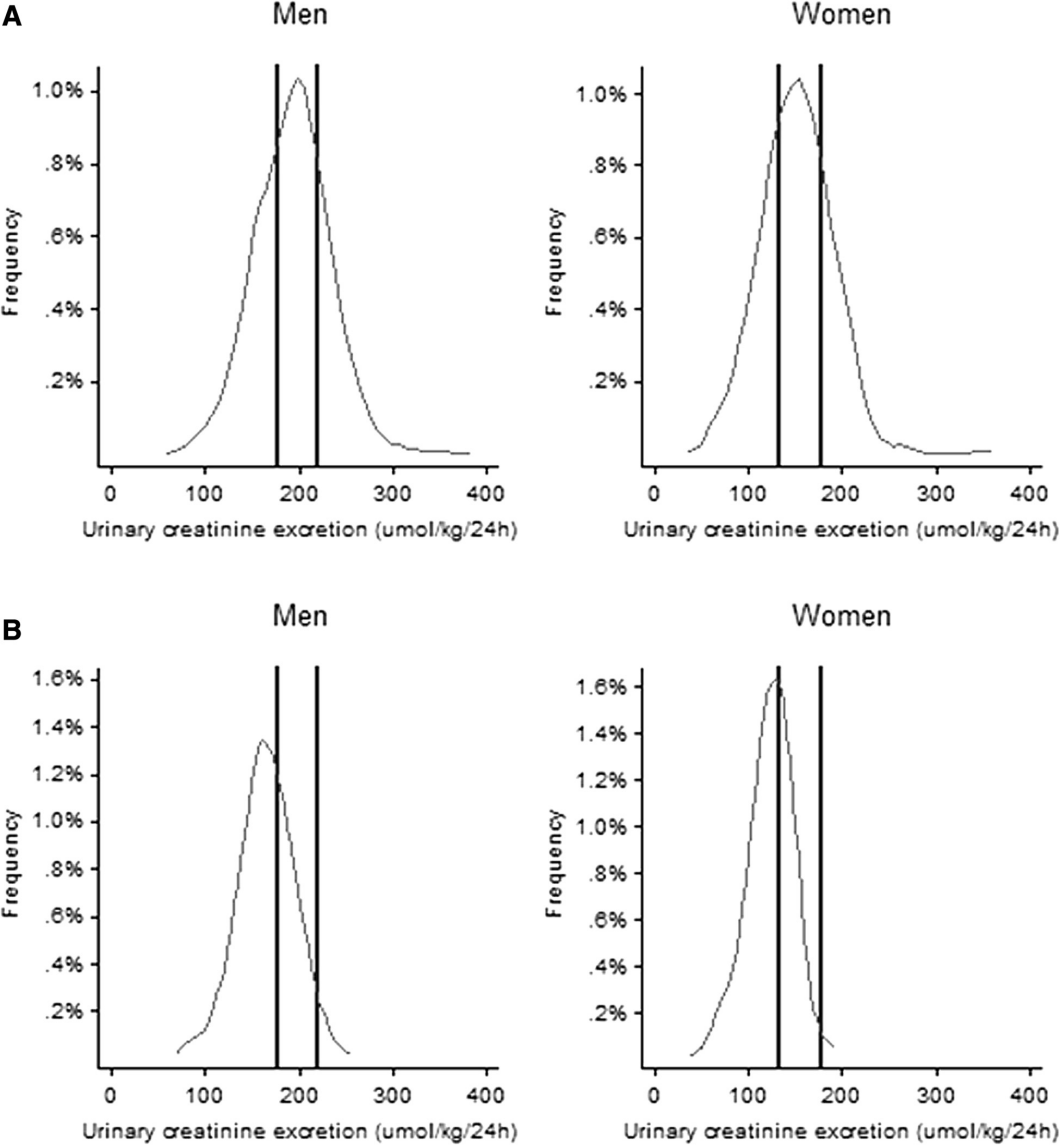
**Distribution of 24-hour urinary creatinine excretion by sex. A)** Whole SSS population and **B)** Population >60 years old. Vertical lines represent the reference interval according to the current reference values of 177 to 221 μmol/kg/24 hours in men and 133 to 177 μmol/kg/24 hours in women. To convert creatinine from μmol to mg, divide by 8.84. SSS: Swiss Survey on Salt.

The mean urinary creatinine excretion in the SSS population was higher in men (193 ± 41 μmol/kg/24 hours) than in women (151 ± 38 μmol/kg/24 hours; *P* <0.001), whereas urinary volume and duration of collection were similar (Table 1). In both sexes, the values of urinary creatinine excretion were normally distributed (Figure 1).

In order to assess reproducibility of urine collection and urinary creatinine excretion, a sub-sample of 49 participants (27 men and 22 women) from seven study centres provided 24-hour urinary collections on two consecutive days. Urinary creatinine excretion was 170 ± 34 μmol/kg/24 hours in the first and 175 ± 38 μmol/kg/24 hours in the second collection (*P* = 0.248). The Pearson’s correlation coefficient for creatinine excretion between the two collections was 0.692 (95% confidence interval (CI) 0.510 to 0.814).

According to the current reference values of 177 to 221 μmol/kg/24 hours in men and 133 to 177 μmol/kg/24 hours in women, 304 of 550 (55.3%) urinary collections in men and 335 of 587 (57.1%) in women were out of range and would, therefore, have been considered as incomplete (32.7%) or over-collected (23.5%) (Figure 1). In people older than 60 years, only 32.9% (99 of 301) of the 24-hour urinary collections agreed with the suggested reference values, whilst 64.8% would have been considered as incomplete and 2.3% as over-collected.

**Figure 2:**
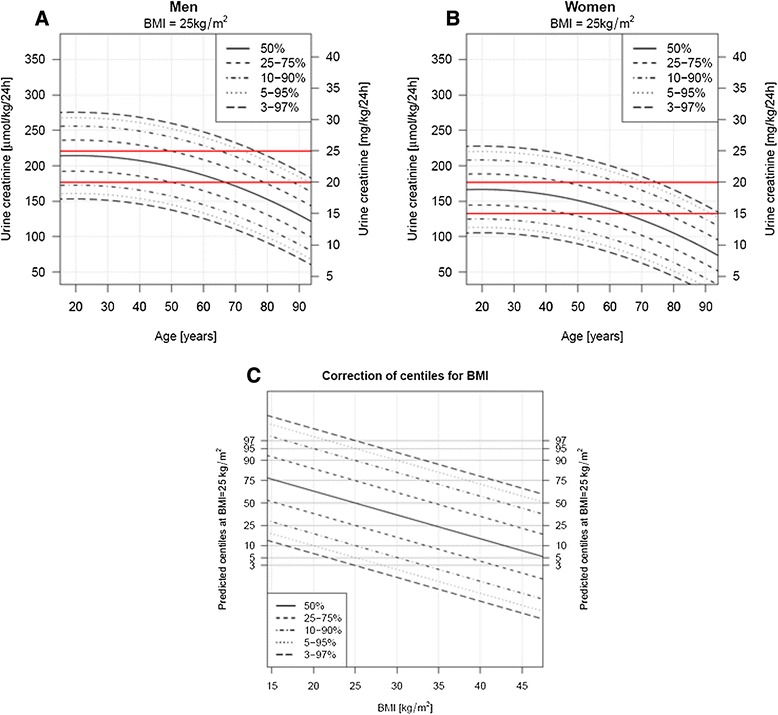
**Normograms of 24-hour urinary creatinine excretion (μmol/kg/24 hours). A)** Men and **B)** Women**.** The dashed lines represent centiles curves of the 24-hour urinary creatinine excretion, according to age and sex, estimated for a BMI of 25 kg/m^2^. Horizontal lines show the current reference values of 177 to 221 μmol/kg/24 hours in men and 133 to 177 μmol/kg/24 hours in women. BMI: body mass index; to convert creatinine from μmol to mg, divide by 8.84. **C)** Correction of centiles obtained in normograms A and B, according to BMI. Application example: estimating the percentile represented by a 24-hour urinary creatinine excretion of 200 μmol/kg/24 hours in a 40-year old man with BMI 35 kg/m^2^: in A, the intersection of age 40 years (on X axis) and urine creatinine 200 μmol/kg/24 hours (on Y axis) corresponds to the 50th percentile. In C, the intersection of BMI 35 kg/m^2^ (on X axis) and the percentile 50 obtained from A (on Y axis) gives a percentile corrected for BMI of 75 (upper dashed line).

We observed a quadratic decline of urinary creatinine excretion (μmol/kg/24 hours) with age in both men and women, with a similar association in both sexes (*P* <0.0001) (Figure 2).

### Modelled reference equation

A regression model with age, sex and BMI as predictor variables was found to provide the best fit of the observed values.

The model applied for the centiles computation is given as follows:

24-hour urinary creatinine excretion (μmol/kg/24 hours) = β_0_ + β_1_*sex + β_2_*BMI + β_3_*age + β_4_*age^2^.

The coefficients of the regression model are reported in Table 2.

**Table 2:** **Coefficients of the regression model used for the centiles computation**

**Variable**	**Parameter**	**Coefficient**	***P*** **-value**
Intercept	β_0_	266.16	<0.001
Sex (if women)	β_1_	−47.71	<0.001
BMI (kg/m^2^)	β_2_	−2.33	<0.001
Age (years)	β_3_	0.66	0.05
Age^2^ (years^2^)	β_4_	−0.017	<0.001

A linear regression model was used to predict the 24-hour urinary creatinine excretion per body weight (expressed in μmol/kg/24 hours).

The standard deviation of the model’s residuals was equal to 32.4.

For example, the predicted 24-hour urinary creatinine excretion for a 40-year old woman (sex = 1) with a BMI of 20 kg/m^2^ is obtained as:

266.16 - 47.71 * 1–2.33 * 20 + 0.66 * 40–0.017 * 40^2^ = 171.1 μmol/kg/24 hours

To get the 95% quantile, we compute 171.1 + Φ(0.95) * 32.4 = 224.2 μmol/kg/24 hours, where Φ(0.95) = 1.64 is the cumulative distribution function of a standard normal.

The inverse transformation allows the estimation of the quantile corresponding to an observation. If we would measure in the same woman a value of 149.6 μmol/kg/24 hours, this would correspond to the quantile given by:

$$ {\Phi}^{-1}\left(\frac{149.6-171.1}{32.4}\right)=0.25 $$

To ease the clinical application of the prediction model at the bedside, we derived two normograms representing the principal centiles curves of the 24-hour urinary creatinine excretion distribution according to age, in both men and women. These centiles curves are estimated for a BMI of 25 kg/m^2^ and a third normogram allows the obtained centile to be corrected according to BMI (Figure 2).

### Validation of the model in the SKIPOGH population

**Figure 3:**
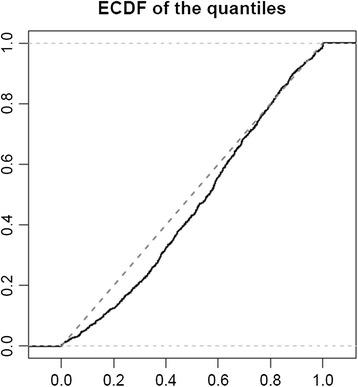
**Validation of the prediction model: cumulative distribution of the predicted quantile values.** ECDF: empirical cumulative distribution function.

We estimated the corresponding quantiles for all observations of the SKIPOGH study in an attempt to validate our prediction model. These centiles followed a uniform distribution, with a Kolmogorov-Smirnov test statistic equal to 0.078 (*P* <0.001). This means that the highest error that we made with our percentile prediction is <11% (Figure 3).

Using the predicted 10th to 90th percentiles as an example of the possible range for adequate collection, 6% of the samples of the SKIPOGH population would have been considered as incomplete and 10% as over-collections. Compared to the current reference values, 416 (42%) samples were reclassified as adequate in the whole population. This proportion increased to 52% when considering only people over 60 years old, where 8 (3%) instead of 150 (58%) samples would have been considered as incomplete and 22 (9%) instead of 13 (5%) as over-collected (Table 3).

**Table 3:** **Adequacy of the 24-hour urinary collection using the current reference values and the new prediction model, in the whole SKIPOGH population and in the elderly patients**

**Classification according to current reference values**	**Classification according to the new prediction model** ^**a**^			**Percentage of reclassified samples**
	**Incomplete**	**Adequate**	**Overcollected**	
	**(<10th centile)**	**(10th to 90th cent)**	**(>90th centile)**	
**Whole population**				**44.2%**
Incomplete	57	247	1^b^	
Adequate	1	406	10	
Overcollected	0	180	92	
**Older population (age >60 years)**				**59.9%**
Incomplete	8	141	1^b^	
Adequate	0	84	10	
Overcollected	0	2	11	

^a^The selection of 10th to 90th as cut-off is arbitrary and should be interpreted as an example of application of the prediction equation; ^b^this observation is from a 90-year-old woman with 24-hour urinary creatinine of 132 μmol/kg/24 hours and BMI 23.8 kg/m^2^. Values represent number of people. Current reference values for adequate 24-hour urinary collection: 177 to 221 μmol/kg (20 to 25 mg/kg) in men and 133 to 177 μmol/kg (15 to 20 mg/kg) in women.

## Discussion

Using two large Swiss population-based studies, we generated and validated a prediction equation for the 24-hour urinary creatinine excretion in the general adult population of European descent. The development of new reference values is warranted, because the current references are poorly representative of the general adult European population and their application would lead us to discard more than half of the 24-hour urinary samples of our two study populations.

The equation proposed herein will provide healthcare providers and researchers with a valuable tool to interpret the completeness of an individual 24-hour urine collection. We additionally developed age- and sex-specific normograms that can be used at the bedside to easily determine where a person fits compared to a reference population.

Timed urine specimens are a keystone of several metabolic evaluations, and as such are commonly used for clinical investigations [27-30] and epidemiological surveys, such as cross-sectional population studies on nutrition [11,12,22,31]. In this context, the identification of over- and under-collection of 24-hour urine samples represents a crucial task to validate and interpret the measured data.

The currently accepted and widely used reference values for 24-hour urinary creatinine excretion of 177 to 221 μmol/kg/24 hours (20 to 25 mg/kg/24 hours) in men and 133 to 177 μmol/kg/24 hours (15 to 20 mg/kg/24 hours) in women rely on observations made in the 1960s and 1970s, and the information on how these ranges were derived from the original descriptions are scarce. In a publication of 1984, Imbembo and Walser [8] reported a linear regression formula for the calculation of creatinine excretion per kilogram according to age and sex, derived from pooling the results of four previous studies published between 1963 and 1976, including 140 to 370 mostly hospitalised people [15-18]. The decline in the urinary creatinine excretion with progressing age is not considered in the current reference values or, at most, mentioned as an expected progressive decline of nearly 50% from the ages of 50 to 90 years.

We questioned the appropriateness of these reference values in a general European population and, applying them to the population of our study, found that more than half of the 24-hour urinary collections would be considered as not valid and two thirds of the urinary collections of participants over 60 years old would be discarded because of suspected over- or under- collection. Although we cannot exclude that the final analysis of our study populations also included some remaining participants with over- or under-collected urinary collections, it seems very unlikely that this would correspond to the numbers obtained when applying classical reference values.

Several factors might explain these differences. First of all, validation was in general not performed in the mentioned studies. To the best of our knowledge, only one previous study [32] validated a creatinine excretion prediction equation in a different population than the derivation population. However, the study from Ix *et al*. [32] was performed on pooled data from different interventional studies, performed on selected patients (mostly diabetic and suffering from chronic kidney disease), limiting, therefore, the applicability of the results to the general population (that is, external validity). Besides, population characteristics have changed. The European population is aging, and the prevalence of obesity is increasing [19-21]. It is, therefore, not surprising that many urinary collections would be suspected of under-collection applying the classical reference values.

Our results should be interpreted in the light of the study’s strengths and limitations.

We used an unselected adult population of European descent with a large sample size to generate a prediction equation for 24-hour urinary creatinine excretion. The equation includes commonly available variables, which are relevant to optimising the estimation of the creatinine excretion. To facilitate the clinical application of our equation at the patient’s bedside, we provided age- and sex-specific normograms and a normogram to correct the estimated percentile for BMI.

The major strength of this study is to have validated the proposed prediction equation in a second independent population-based sample from the same country. Environmental conditions, including socio-economic level and nutritional habits are, therefore, similar in both studies.

One major limitation of our study design is the absence of a gold standard to define the correctness of the urine collection. We could have used para-aminobenzoic acid (PABA) as a possible reference comparator, using its integral urinary excretion after oral intake as an indicator of the 24-hour urinary collection completeness. However, this method has some important limitations, including not permitting the identification of urine over-collections, having a reduced reliability in older people and being dependent on the compliance of the participants with the PABA intake, a potentially problematic issue in large scale population-based studies [10,33]. We consider that the finding of a normal distribution of the urinary creatinine excretion in a large population-based sample and the reproducibility of the values in the small subsample with repeated urine collections, support the overall correctness of the urine collections.

Another limitation, linked to the statistical approach used to generate the prediction equation, is that the predicted values are shrunk towards the mean, thus the variability of the prediction is smaller than the original variability. This could limit the reliability of predictions at the individual-level.

Given the low number of participants in the group older than 75 years and the fact that older people often suffer from sarcopenia, the proposed normograms should be used with caution in older people.

It should finally be underlined that the presented prediction equation has been developed on people without chronic kidney disease, limiting its utilisation in this population unless further validated.

## Conclusions

Although widely used as an accuracy marker of 24-hour urine collections, the current reference values for 24-hour urinary creatinine excretion are poorly representative of the general adult European population. Their systematic application would lead us to discard - because of suspected over- or under-collection - more than half of the 24-hour urinary collections in the whole population and two thirds of the urinary collections of people over 60 years old in the two population-based samples analysed in this study.

We propose a validated prediction equation for 24-hour urinary creatinine excretion in the general European population and a few derived normograms, based on readily available variables, which should help healthcare providers and researchers interpreting the completeness of an individual 24-hour urinary collection in daily clinical practice and in epidemiological population studies.

## Abbreviations

BMI: body mass index
CKD-EPI: Chronic Kidney Disease Epidemiology Collaboration
eGFR: estimated glomerular filtration rate
PABA: para-aminobenzoic acid
SKIPOGH: Swiss Kidney Project on Genes in Hypertension
SSS: Swiss Survey on Salt
